# Craniofacial implants in a failed autologous reconstruction of microtia: a case report

**DOI:** 10.1186/s40729-021-00337-8

**Published:** 2021-06-21

**Authors:** Vladimir Frias

**Affiliations:** Department of Oral Oncology, Roswell Park Comprehensive Cancer Center, Elm & Carlton Streets, Buffalo, NY 14203 USA

**Keywords:** Microtia, Craniofacial implants, Auricular prosthesis

## Abstract

Plastic surgical reconstruction is considered to be the gold standard for the repair of microtia as the results are permanent and constructed from the patient’s own tissue; however, the multiple surgeries required and the difficulty in attaining adequate cosmetic results often result in patients choosing a prosthesis as a long-term rehabilitation. Advances in osseointegration in the craniofacial region have improved the outcomes with auricular prosthetics by providing a reliable method of attachment of the prosthesis and increasing patient acceptance. A case presentation illustrates the results of both treatment modalities and examines the outcomes on the same patient.

## Introduction

Although the word microtia (micro-otia) literally translates as “small ear,” the clinical condition presents as anything from an ear that presents with minor deformities but with all major landmarks present, to a severely malformed ear that presents with few identifiable landmarks [1]. The remnants of the auricle may be displaced, and the condition is often associated with aural atresia, hearing loss, and craniofacial syndromes [2]. Risk factors for developing microtia include embryonic vascular disruption; environmental factors such as maternal age, illness, or medication; or genetic pathways [3].

Options for the rehabilitation of microtia have included plastic surgical reconstruction and craniofacial prosthetics with or without the use of osseointegrated implant retention mechanisms [4]. The use of autogenous rib cartilage for the reconstruction was described by Tanzer [5], and his method has formed the basis for most current surgical options. Two widely used and successful techniques based on autogenous rib grafts have been proposed by Brent [6, 7] and Nagata [8]. The Brent technique is based on the original surgical approach used by Tanzer but uses four surgical stages instead of the original six. The procedures include the fabrication of the auricular framework with costal cartilage followed by transposition of the lobule, elevation of the framework, and reconstruction of the tragus. The number of stages needed for reconstruction has often been cited as a deficiency of the technique as, in practice, the number of surgical procedures including revision procedures can often reach seven or eight. Although the Nagata technique also uses autogenous rib cartilage, it differs from the Brent technique by proposing two stages which combine framework harvesting and contouring, tragus reconstruction, and lobule transposition in one procedure followed by framework elevation at the second stage. This reduces the number of surgeries significantly; however, the procedure has been shown to result in an increased rate of complications including flap necrosis, framework extrusion or resorption, and increased donor site complications. Even in successfully treated cases with either technique, there is often an esthetic compromise resulting from the lack of definition of the concha and surrounding structures.

Advances in biomedical engineering may eliminate some of the problems with the surgical reconstruction of auricular defects. Current experiments are focused on the creation of tissue-engineered cartilage that has improved elasticity compared to harvested rib cartilage [9]. The advantage of this technique is that it allows a precise framework to be created in the laboratory rather than sculpting the cartilage in the operating room, and it also reduces the surgical invasiveness of the reconstruction [10].

An alternative approach to the treatment of microtia has been to use a prosthetic material to replace the missing or malformed portions of the ear. The use of artificial prostheses to restore facial structures has been recorded since ancient times [11], and the use of leather, fabrics, clay, and metal as prosthetic materials and retention mechanisms have all been reported [12]. The improvement in dental materials in the twentieth century allowed for increasingly realistic prosthetics; however, many of the newer materials did not possess the durability required of a long-term prosthetic restoration, nor a reliable method to attach it to a defect. The evolution of advanced silicone elastomers and the introduction of osseointegrated craniofacial implants [13] as a method of prosthetic attachment have improved the issues with durability, cosmetics, and retention. Because of the favorable conditions at the mastoid region, the success rate of osseointegrated implants retaining auricular prostheses or bone-anchored hearing aids has been exceptionally high with success rates above 95% [14].

The use of craniofacial prosthetics for the rehabilitation of microtia often results in superior esthetic results with a minimal number of surgical procedures; however, there are several marked deficiencies with this approach, chief amongst which is the removable nature of the reconstruction. The daily maintenance procedures which include careful debridement of the supporting structures with a brush and detergent can also be complicated for patients with limited mobility [15]. Despite the high success rates of auricular implants, there are multiple issues that arise with the tissue and the prostheses themselves. Local tissue reactions from erythema through granulation tissue have been noted [16]. Studies have also shown that the remake rate of an auricular prosthesis due to poor fit or discoloration is approximately 14 months. Other complications that may arise are loss of retention of the attachment clips, loosening of bar screw or abutments, separation of the retention clips or acrylic base from the silicone, and rupture of the silicone [17]. Also important is the fact that the removal of residual tissues for the placement of osseointegrated craniofacial prostheses often eliminates the possibility of future plastic surgery reconstruction.

In many cases, the ultimate choice of rehabilitation is determined by the experiences and the expertise of the treating physicians, and because of the limited number of rehabilitation centers, the cost, and the time involved in either reconstructive method, many patients choose their treatment options by what is available locally rather than explore all available treatments. A careful consideration of the position and size of the auricular remnants and the long-term needs and desires of the patient should be included in the decision-making process prior to the selection and implementation of a therapeutic protocol. The following case presentation illustrates the different results attained in a patient who had both a plastic surgical reconstruction followed by an osseointegrated craniofacial prosthesis.

## Clinical report

**Fig. 1:**
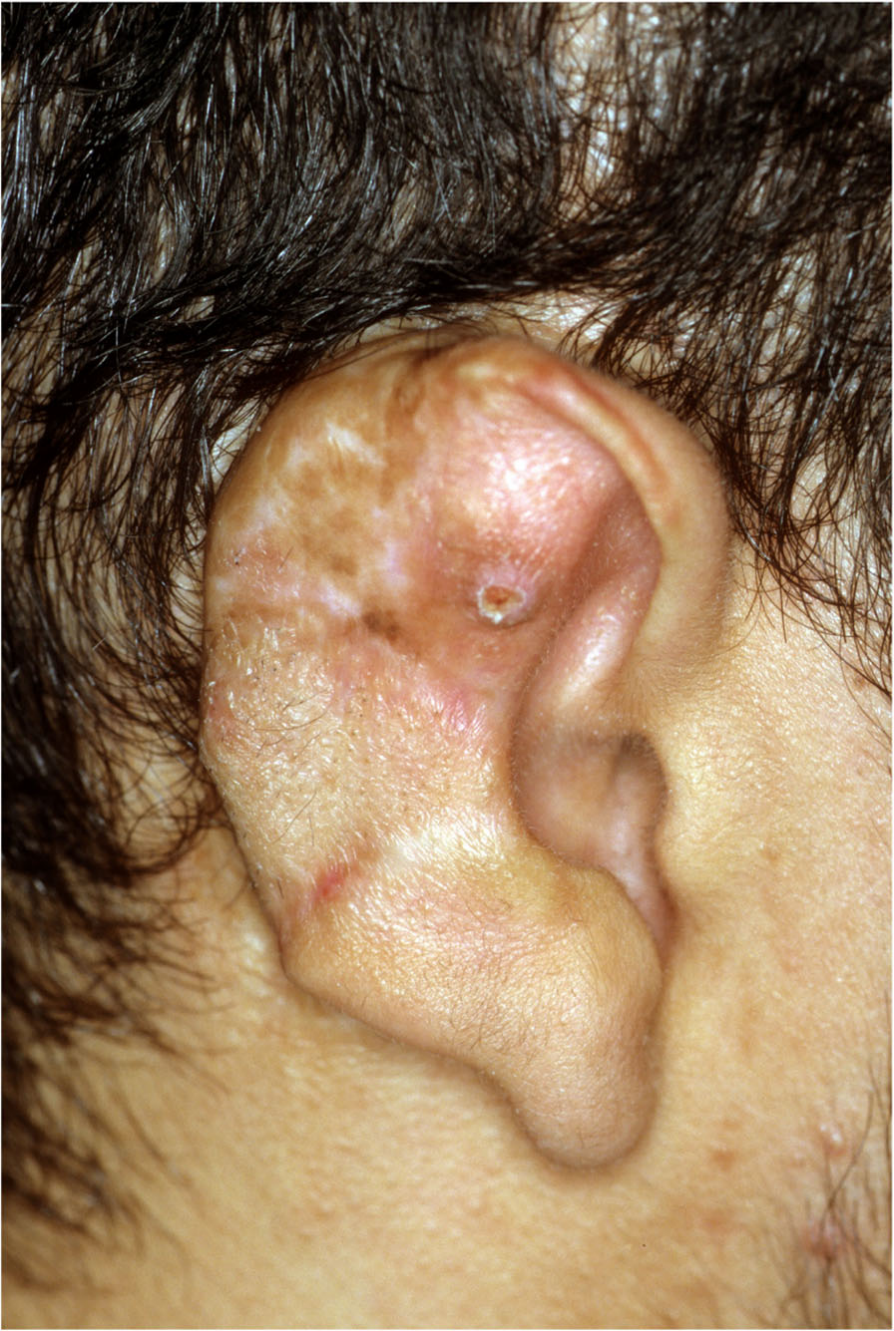
Right auricle, post-reconstruction

**Fig. 2:**
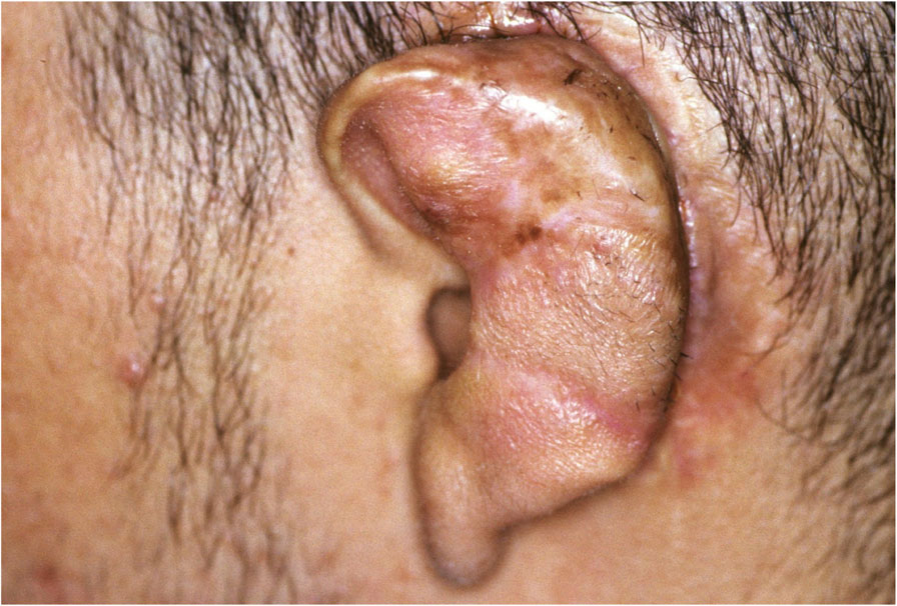
Left auricle, post-reconstruction

A 19-year-old man presented with a history which included bilateral congenital defects that had been surgically reconstructed over the past 6 years. Surgical intervention was begun on the right ear at age 13, and the surgical procedures were deemed complete after six procedures (Fig. 1). Surgical intervention for the left ear was begun, and after a total of seven procedures, the surgical course of treatment was completed (Fig. 2). The patient was especially dissatisfied with the appearance of his left ear, and his plastic surgeon gave him the option for an implant-retained craniofacial prosthesis.

**Fig. 3:**
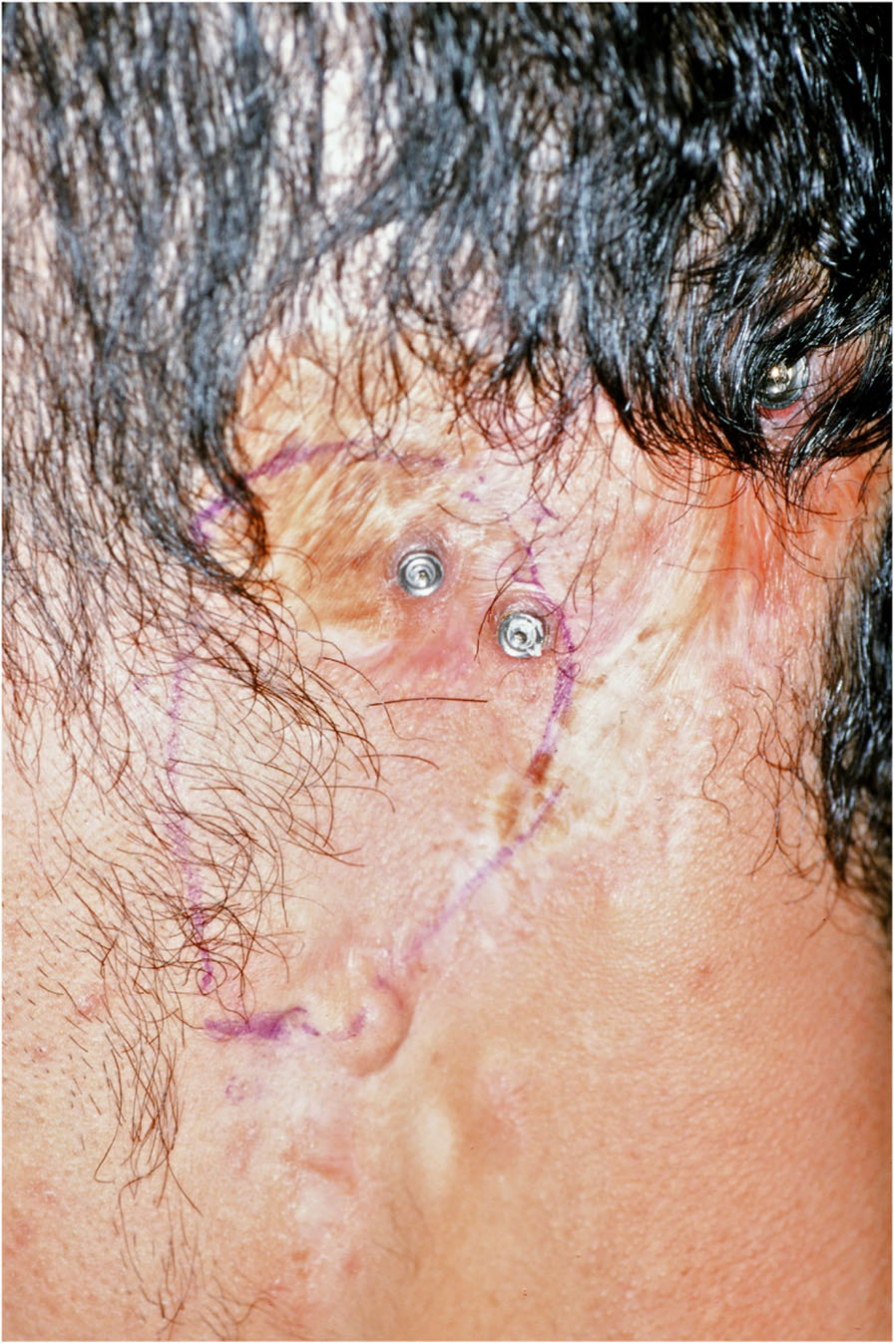
Implants with abutments attached

**Fig. 4:**
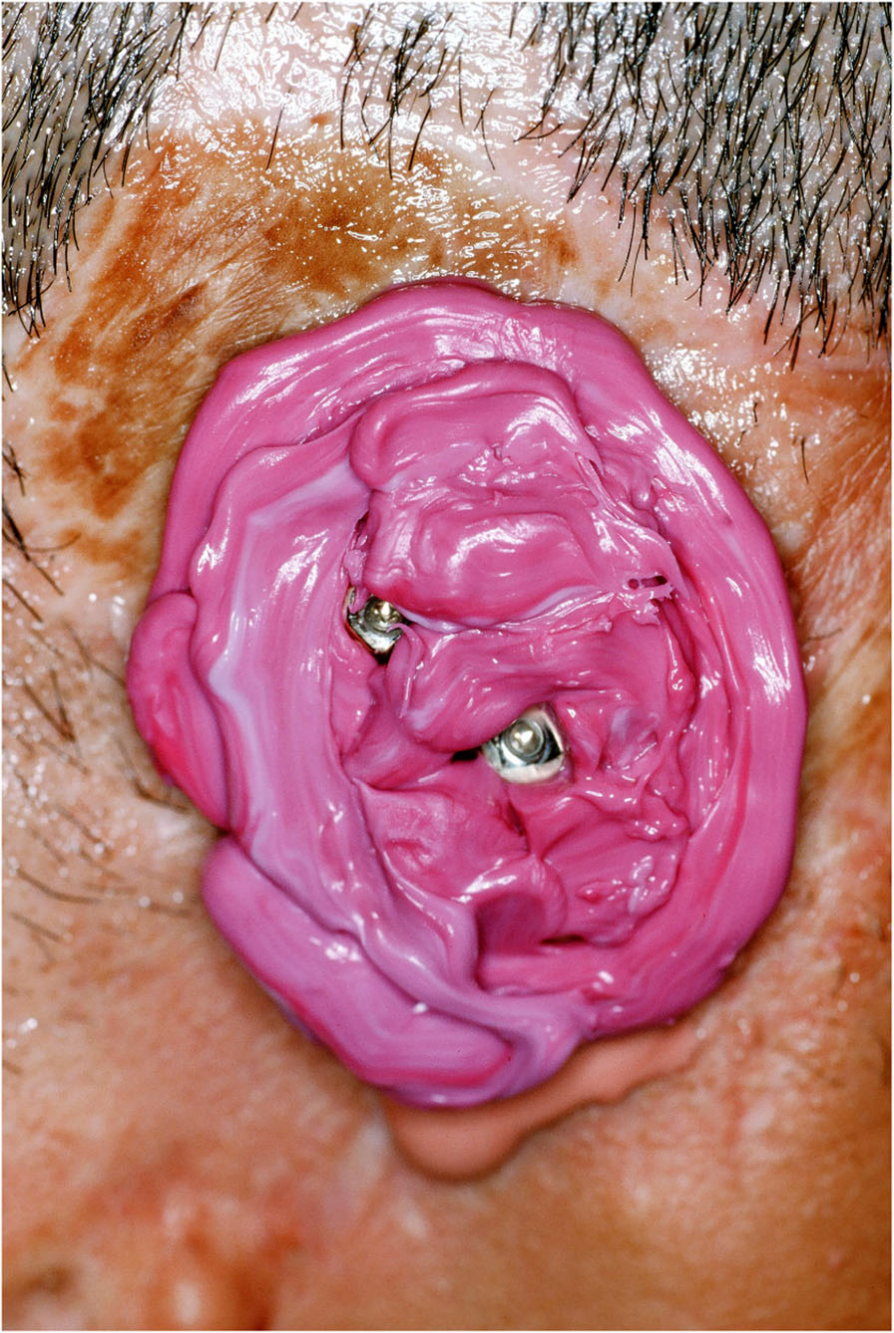
Pickup of the impression copings in silicone

**Fig. 5:**
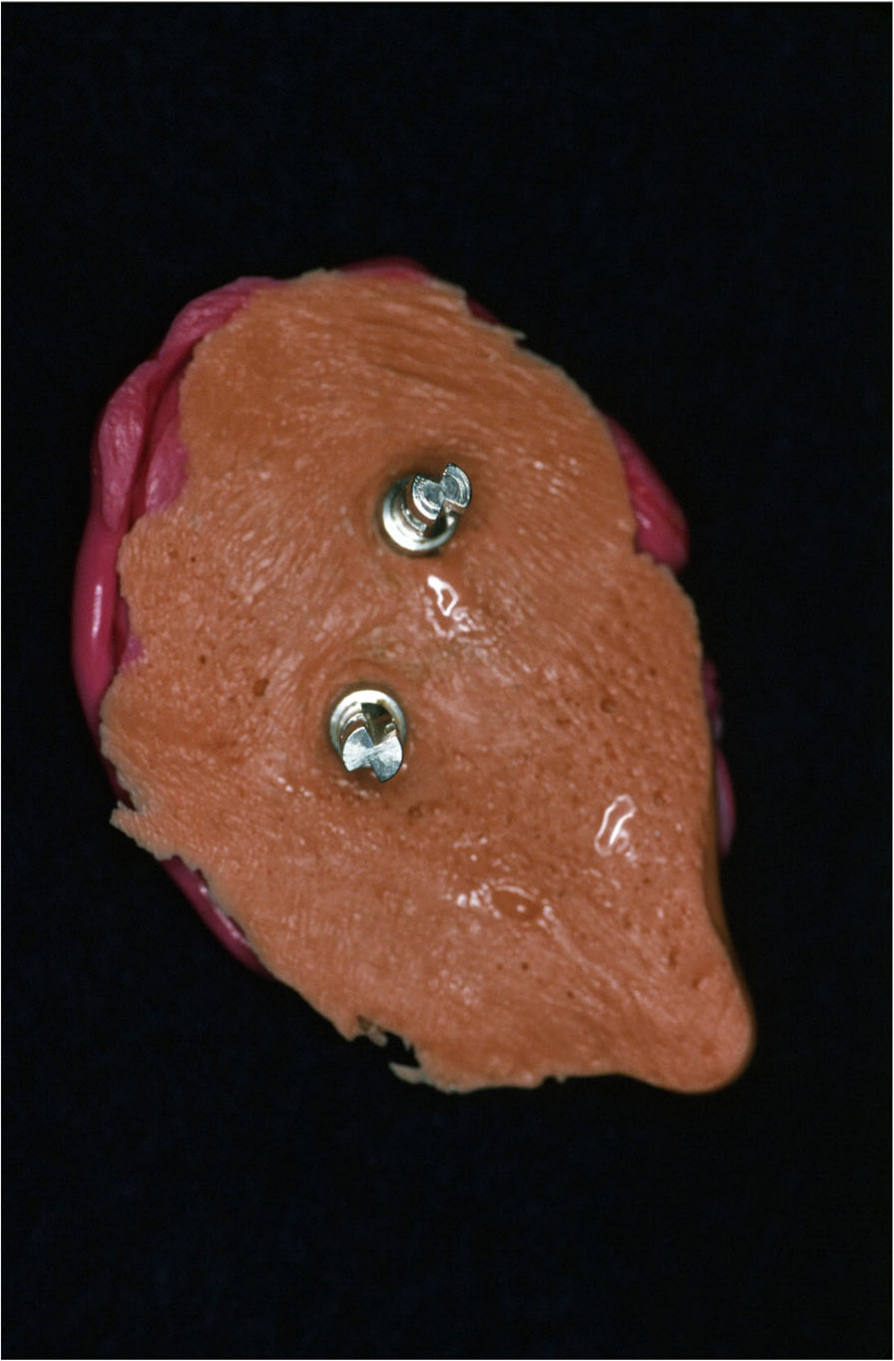
Abutment analogs connected to the impression

**Fig. 6:**
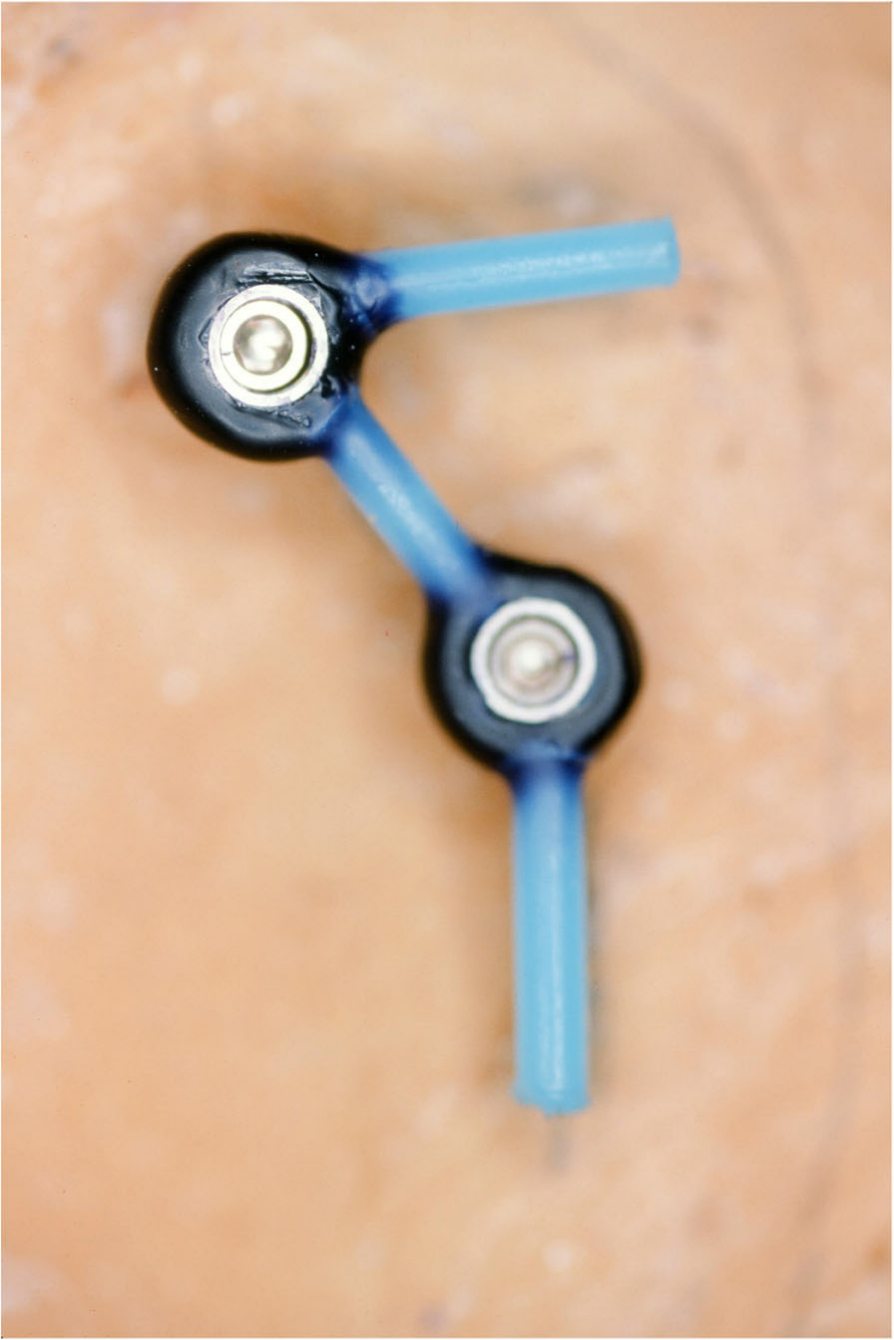
Framework designed to fit under the helix

**Fig. 7:**
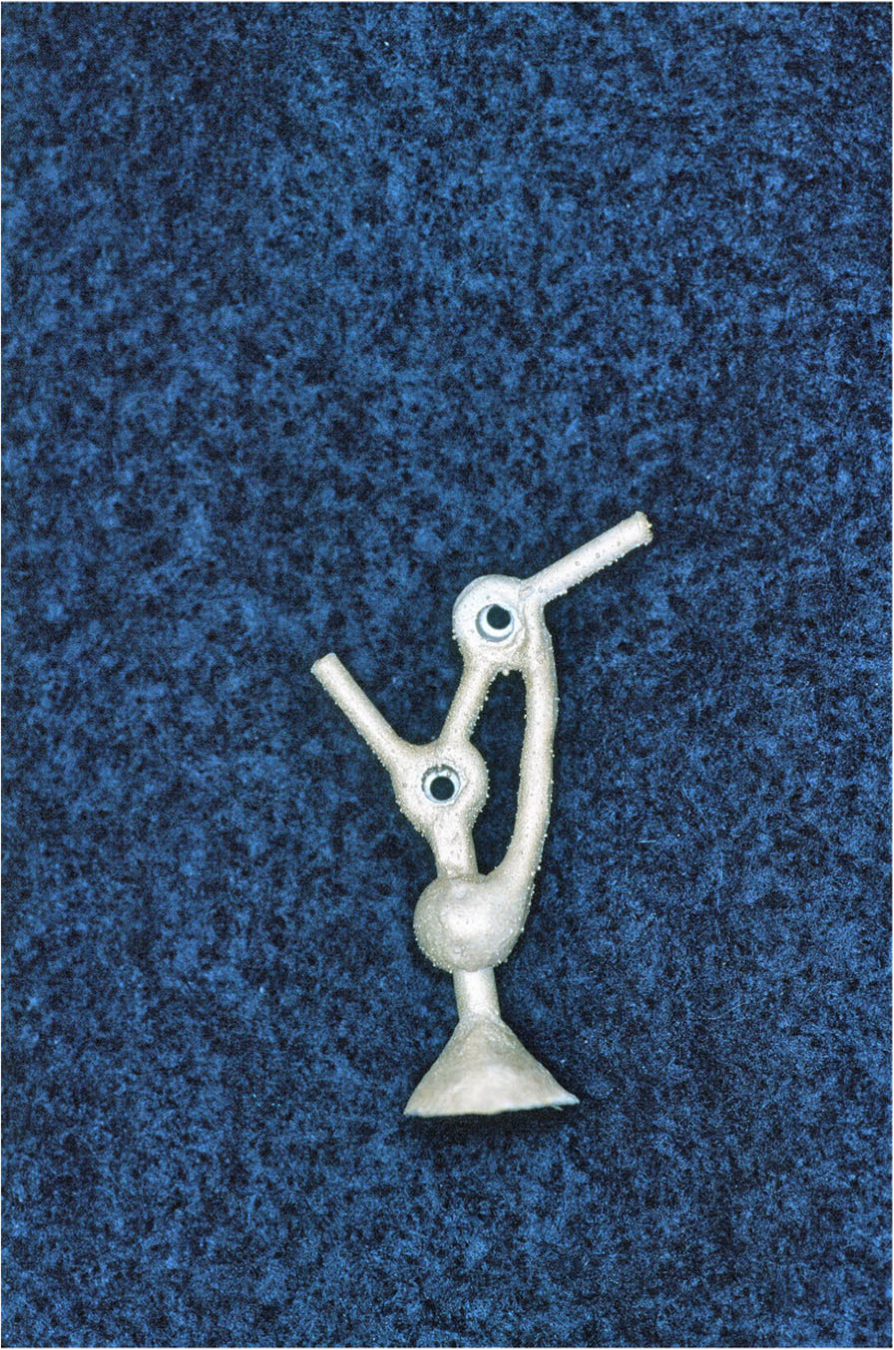
Retention bar cast in type III gold alloy

**Fig. 8:**
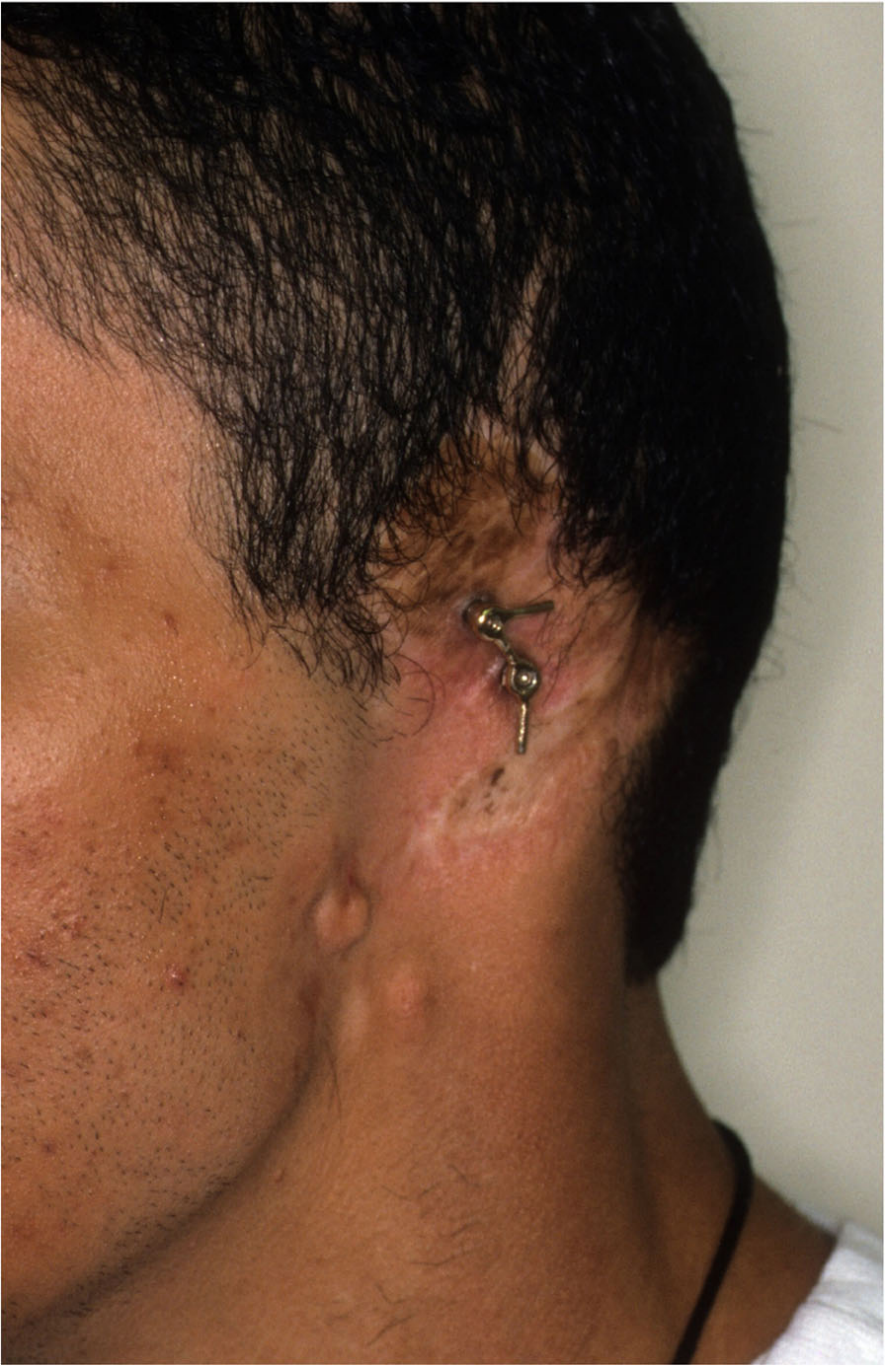
Bar framework attached to implants

**Fig. 9:**
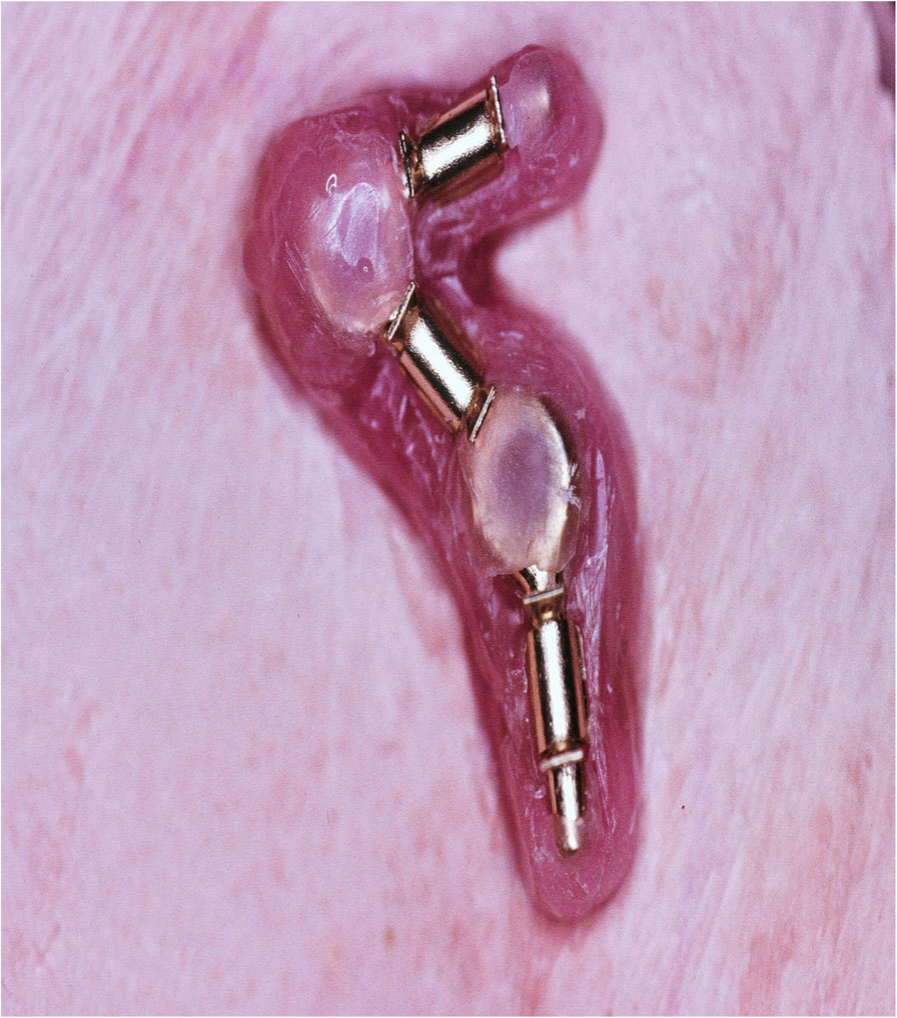
Retention clips blocked out on the bar

**Fig. 10:**
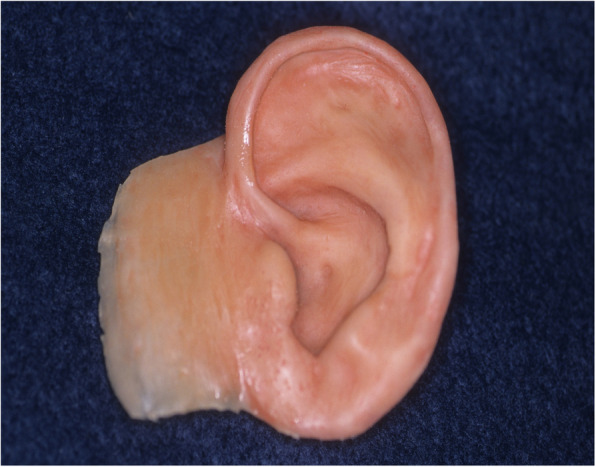
Processed intrinsically colored prosthesis with tissue flange

**Fig. 11:**
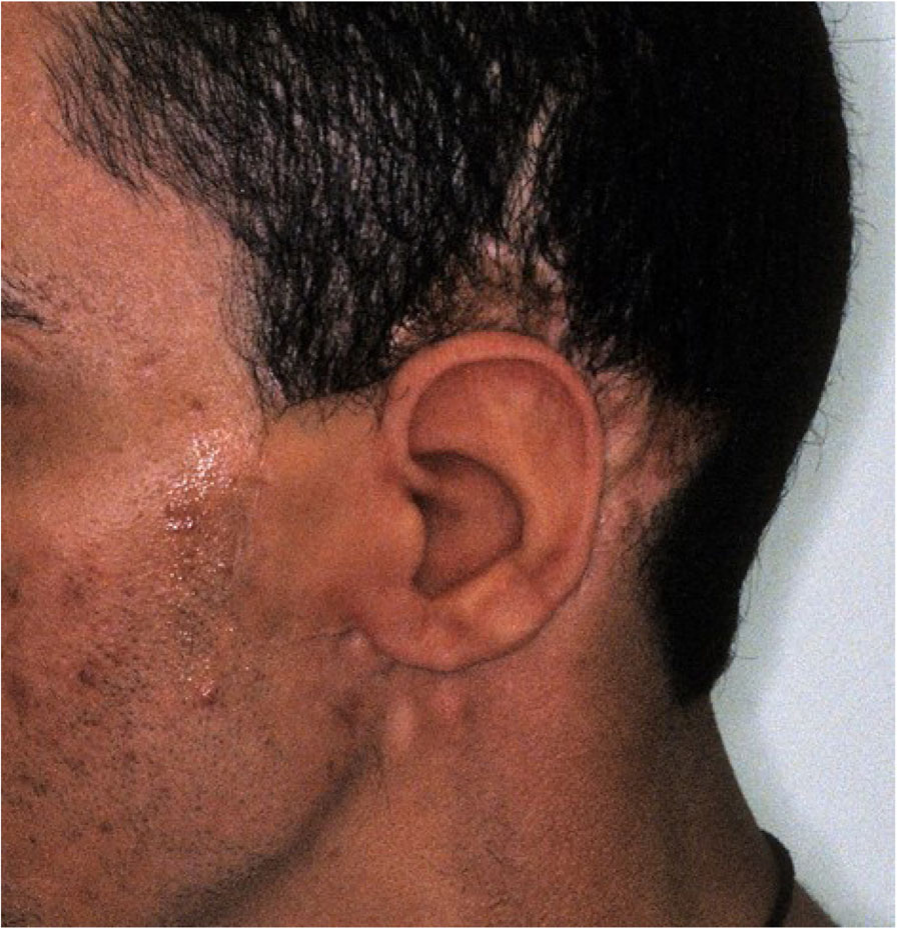
Lateral view of the completed prosthesis

An impression of the reconstructed ear was made with irreversible hydrocolloid [Jeltrate, Dentsply Caulk, Milford DE], and a wax pattern of an idealized auricle was created and approved by the patient. In cases where a normal ear is present, a copy of the opposing auricle can be created in a mirror image from digital data; however, since the patient was not satisfied with the esthetics of his contra-lateral ear, the pattern for the new auricle was created by hand. The wax pattern was replicated in acrylic to create a surgical guide for implant placement. Since the meatus would be the only remaining anatomical landmark after removal of the reconstructed ear, the guide was scored along the inferior ala-superior meatal line to allow for ease of orientation. The removal of the auricular reconstruction and placement of the implants were performed at the same surgical appointment. Two VXI 300 implants [Vistafix 3, Cochlear Americas, Centennial, CO] were inserted into the mastoid area at positions which would allow the placement of the framework under the antihelix. The Vistafix 3 implant system is specifically designed for craniofacial applications, and the implants are available in lengths of 3mm and 4mm with a diameter of 4.5mm. The length of implant chosen is determined from a pre-operative medical CT which allows bone measurement as well as clinical confirmation of osseous housing during surgery. Cover screws were placed, and the implants were submerged and allowed to heal for 3 months. On completion of healing, the implants were uncovered and multi-unit abutments were attached. The outline of the wax pattern was traced to confirm positioning of the abutments and for framework design (Fig. 3). Impression copings were attached and connected with pattern resin (GC pattern resin, GC America, Alsip, IL). The impression was made with injectable low-viscosity vinyl polysiloxane (Reprosil, Dentsply Caulk, Milford DE) and reinforced with putty silicone (Figs. 4 and 5). The impression was boxed and poured according to standard techniques. A pattern for the retention bar was created by connecting plastic bar patterns (CBS bar system, Attachments Intl, San Mateo, CA) to the prefabricated gold cylinders screwed to the abutments (Fig. 6). A silicone index for the helix and anti-helical portions of the auricle was created, and the bar extensions were adjusted to fit under the contours of the wax pattern of the auricle. The bar pattern was invested and cast in type III dental gold (Fig. 7), then polished and verified (Fig. 8). Matching retention clips were placed on the bar and connected with acrylic resin (Fig. 9). Retention nodules were placed on the acrylic framework to aid in adhesion to the silicone overlay. The wax pattern for the final prosthesis was created using the original wax pattern as a guide, and the bar and wax pattern were tried in and verified. The wax anterior to the tragus was extended anteriorly to prevent a gap from developing between the prosthesis and skin surface on jaw opening (Fig. 10). The patient’s skin shade was matched using the FI-SK skin coloration system (Factor II Inc., Lakeside, AZ). The wax pattern was finished and invested, and the prosthesis was processed in silicone (A2000, Factor II Inc., Lakeside, AZ) using conventional techniques. The prosthesis was extrinsically colored and sealed before delivery to the patient (Fig. 11). The sequence of procedures is delineated in Table 1.

**Table 1:** Sequence of treatment procedures

Impression of auricular remnants Creation of ideal auricular contours Creation of surgical guide Placement of implants Abutment connection Pickup of abutment copings Fabrication of retentive bar Fabrication of clip housing Final auricular wax pattern Mold creation and silicone process	

The patient was instructed in home care, and a recall system was set up. He was followed up annually for 3 years prior to moving away. Mild erythema and buildup of sebum were noted at the first recall which was eliminated with improved hygiene of the area using a soft bristle brush and 3% hydrogen peroxide. The issue was evaluated by visual and tactile examination. No evidence of swelling, bleeding, or suppuration was noted. The prosthesis was remade at 18 months due to the deterioration of the silicone flap. No complications were noted with the implants, abutments, or bar and clip framework. The tissue reaction and remake rates are similar to published studies [18].

## Conclusions

A clinical case presentation illustrates the treatment alternatives that face the patient suffering from microtia. Since the surgical reconstruction of microtia is often begun at a young age, parents are usually faced with a decision to undergo a series of operations for the reconstruction of the auricle or to have craniofacial implants placed. The use of one or the other treatment modality is often dependent on the skills and training of the surgical staff as auricular reconstruction is a highly specialized procedure which is not available at all medical centers. Although in cases amenable to surgical reconstruction, it is the option that should be considered first, some parents may decide not to subject their children to the multiple surgeries involved in auricular reconstruction. Other reasons for choosing an osseointegrated prosthesis are a significant deformity, burns, or a medical condition that precludes surgical reconstruction. In other cases, the placement of implants for a bone-anchored hearing aid is a good opportunity to insert additional implants for a craniofacial prosthesis. The final reason for the use of osseointegrated prostheses is the failure of a surgical reconstruction due to extrusion, resorption, or a poor cosmetic outcome. The clinical presentation shows how even multiple surgeries by an experienced surgical team can result in less than acceptable cosmetic results and an osseointegrated prosthesis can serve as a viable alternative.

## Data Availability

Not applicable.

## References

[1] Weerda H (1988). Classification of congenital deformities of the auricle. Facial Plast Surg.

[2] Bly RA, Bhrany AD, Murakami CS, Sie KCY (2016). Microtia Reconstruction. Facial Plast Surg Clin North Am.

[3] Gendron C, Schwentker A, Van Aalst JA (2016). Genetic advances in the understanding of microtia. J Pediatr Genet.

[4] Wilkes GH, Wong J, Guilfoyle R (2014). Microtia reconstruction. Plast Reconstr Surg.

[5] Tanzer RC (1959). Total reconstruction of the external ear. Plast Reconstr Surg.

[6] Brent B (1980). The correction of microtia with autogenous cartilage grafts: I. The classic deformity. Plast Reconstr Surg.

[7] Brent B (1980). The correction of microtia with autogenous cartilage grafts: II. Atypical and complex deformities. Plast Reconstr Surg.

[8] Nagata S (1993). A new method for total reconstruction of the auricle for microtia. Plast Reconstr Surg.

[9] Pappa AK, Caballero M (2014). Biochemical properties of tissue-engineered cartilage. J Craniofac Surg.

[10] Hwang CM, Lee BK (2014). Auricular reconstruction using tissue-engineered alloplastic implants for improved clinical outcomes. Plast Reconstr Surg.

[11] Reisberg DJ, Habakuk SW (1990). A history of facial and ocular prosthetics. Adv Opth Plast Reconstr Surg.

[12] Schaaf NG (1975). Materials in maxillofacial prosthetics. Dent Clin Nort Am.

[13] Tjellstrom A, Yontchev E (1985). Five years’ experience with bone-anchored auricular prostheses. Otolaryngol Head Neck Surg.

[14] Jacobsson M, Tjellstrom A (1992). A retrospective study of osseointegrated skin-penetrating titanium fixtures used for retaining facial prostheses. Int J Oral Maxillofac Implants.

[15] Allen PF, Watson G (2000). Peri-implant soft tissue maintenance in patients with craniofacial implant retained prostheses. Int J Oral Maxillofac Surg.

[16] Nishimura RD, Roumanas E (1995). Auricular prostheses and osseointegrated implants: UCLA experience. J Prosthet Dent.

[17] Karacoca S, Aydin C (2010). Retrospective study of treatment outcomes with implant-retained extraoral prostheses: survival rates and prosthetic complications. J Prosthet Dent.

[18] Aydin C, Karakoca S (2008). Implant retained auricular prostheses: an assessment of implant success and prosthetic complications. Int J Prosthodont.

